# A Review of Bioactive Components and Pharmacological Effects of *Ganoderma lucidum*

**DOI:** 10.1002/fsn3.70623

**Published:** 2025-07-13

**Authors:** Sizhu Ren, Hua Liu, Qing Sang, Mengqi Lu, Qing Gao, Wenjie Chen

**Affiliations:** ^1^ Langfang Normal University College of Life Sciences Langfang Hebei Province People's Republic of China; ^2^ Technical Innovation Center for Utilization of Edible and Medicinal Fungi in Hebei Province Langfang People's Republic of China; ^3^ Fushiping Shaohua Agricultural Development Co., Ltd Baoding Hebei People's Republic of China

**Keywords:** anticancer activities, bioactive compounds, *Ganoderma lucidum*, immune responses, medicinal herb

## Abstract

*Ganoderma lucidum*, a distinguished traditional medicinal herb, has long been recognized for its purported benefits in promoting physical well‐being, enhancing stamina, and boosting vitality. However, the mechanistic underpinnings of its antitumor activities and other therapeutic effects remain incompletely understood. Historically, the activation of host immune responses was widely regarded as the sole mechanism by which *G. lucidum* exerts its anticancer effects. Recent advancements, however, have revealed that its antitumor mechanisms extend beyond immune modulation to include induction of cell differentiation, inhibition of angiogenesis, and other molecular pathways. Despite its established status as a functional food and potential therapeutic agent, *G. lucidum* remains an underexplored resource in modern nutraceutical and pharmaceutical research. Notably, few bioactive compounds derived from this mushroom have been clinically validated as superior to existing therapies for disease treatment. This review systematically summarizes the primary bioactive components of *G. lucidum*, including polysaccharides, triterpenoids, peptides/proteins, and sterols and discusses their multifaceted health benefits. Additionally, critical insights into optimizing its industrial applications and evaluating its safety profile are highlighted, emphasizing the need for further research to translate its traditional uses into evidence‐based interventions.

## Graphical abstract

The active substances and medicinal effects of the medicinal mushroom *Ganoderma lucidum* were reviewed.

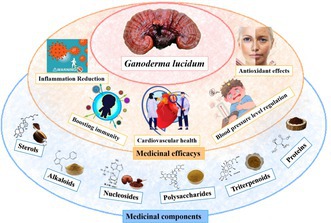

## 1 Introduction

*Ganoderma lucidum* (GL), a macrofungus with medicinal and edible properties, has many different active substances and pharmacological effects, which have received the public's attention (Cen et al. 2024; Yuan et al. 2020). It can moisten the lungs, ensure smoother breaths, and relieve cough. Besides, its body‐fruiting and sporangium powder have effects on immune regulation, liver protection and detoxification, anti‐allergy activities, antitumor activities, treatment of inflammation, and prevention and treatment of diabetes (Wang et al. 2020). The portion of *G. lucidum* is grown on trees, rotten wood, or culture substrates and forms a fruiting body, which consists of the cap and stalk (Thiribhuvanamala and Krishnamoorthy 2021). Different varieties of *G. lucidum* and different growing environments directly affect its medicinal ingredients. Recently, the chemical composition, antioxidant, and antiproliferative activities of different kinds of *G. lucidum* were compared and analyzed, and the results showed that there was a close correlation between these abilities and the active ingredients (Saltarelli et al. 2015). The spores of *G. lucidum* play the reproductive function and germinate mycelia once falling into a suitable growth environment, such as rotten wood (Ura et al. 2016). As the germinated mycelium passes through several stages and reaches a cell age, the nutrients in the mycelium accumulate to a certain period, and it begins to twist to form a fruiting body (Moore et al. 2008). When *G. lucidum* is young and tender, it is fleshy and can be eaten like ordinary mushrooms; with the passage of time, it will gradually lignify, and it is unfavorable for eating as it approaches maturity but has high medicinal value. By that time, *G. lucidum* has the highest content of internal medicinal ingredients. Thereinto, polysaccharides, triterpenoids, trace elements, etc., are the most important bioactive components. These bioactive components are used to cure many diseases. Numerous studies have demonstrated the ability of *G. lucidum* and its active components to regulate gut flora. However, a systematic review of this mechanism is currently lacking.

Driven by the good developmental potential and favorable environment at this stage, *G. lucidum* is recognized as a precious large fungus with medicinal and nutritional healthcare values. Especially in recent years, *G. lucidum* spores have been increasingly perceived as a nutraceutical and functional food. When *G. lucidum* is about to reach full maturity, the ganoderma spores will be ejected. However, because of the existence of a double‐layered sporoderm covered by chitin, the nutritional and medicinal components in sporangium powder are difficult to digest and degrade. Meanwhile, the active ingredients such as triterpenes, alkaloids, and polysaccharides in the spores cannot be released in time, so the wall‐breaking treatment of *G. lucidum* spore is particularly important (Xia et al. 2023). *G. lucidum* spore integrates the essence of *G. lucidum*, and its active components are more easily absorbed by the human body after breaking the wall. The types and contents of active components are higher than those of its fruiting body and mycelium. Sporoderm‐removed *G. lucidum* spore powder processes 1.81% polysaccharide and 1.31% triterpene acid, which is the most important medicinal ingredient in all *G. lucidum* products. Wu et al. (2023) revealed the anticancer mechanism of *G. lucidum* spore powder. Because the *G. lucidum* spore can induce Fe^2+^ influx of cells, accumulating the production of lipid peroxides and ROS. Not only that, the mitochondrial volume and its internal mitochondrial ridges under the action of *G. lucidum* spore were both equally decreased, which can lead to impaired mitochondrial function and trigger ferroptosis. *G. lucidum* has become the most well‐explored and product‐developed medicinal fungi for its numerous bioactive substances and extensive pharmacological effects in recent years.

This paper reviews the latest research results of *G. lucidum* and aims to summarize advancements in the context of studies on the active ingredients and medicinal efficacy of *G. lucidum*. By analyzing case analysis, the fundamental mechanisms could be better understood, which can provide data and research experience that may be useful for future disease prevention and its related basic research on *G. lucidum*.

## 2 Reactive Ingredients

### 2.1 Ganoderan

Ganoderan is formed by the condensation of monosaccharides, and the sugar components of Ganoderan are D‐glucose, D‐mannose, D‐xylose, L‐arabinose, and L‐rhamnose. The main chain of Ganoderan consists of β‐D‐Glcp and some α‐D‐Galp, which will have antioxidant activity if the side chain has β‐D‐Glcp, and it will have immunomodulation activity if the side chain has α‐L‐Fucp (Lu et al. 2020). Ganoderans in the fruit body (10^3^ and 10^6^ Da) are a little smaller than the ganoderans in the spores and mycelia (10^5^–10^6^ Da) (Wu, Zhang, Peng, et al. 2024). Wen et al. (2016) explored the correlation between its polysaccharide formation and environmental parameters. The polysaccharide concentration reaches 1.6 mg/mL when using the dextrose‐ammonium chloride medium. Besides, proper agitation and aeration can affect the polysaccharide yield, although a higher shear rate in the fermenter can improve the mixing efficiency and polysaccharide release but also destroy mycelium growth. Zhao et al. (2010) extracted *G. lucidum* polysaccharides after filtration, chromatography, and purification, determined the molecular weight and ultraviolet and infrared spectra of the polysaccharide unit for its characterization, and measured the activity of the polysaccharide against macrophages and in vitro human breast cancer cells, which revealed that *G. lucidum* polysaccharide can improve macrophage proliferation and inhibit cancer cells.

Cao and Lin (2002) studied the regulatory effects of *G. lucidum* polysaccharides on the maturation and function of in vitro cultured bone marrow‐derived DCs of mice. They found that the polysaccharides could enhance the coexpression of CD11c and I‐A/I‐E molecules on the surface of DCs, which can promote the expression of interleukin (IL)‐12 p40 mRNA in DCs but also increase the level of IL‐12 in the culture supernatant. Moreover, *G. lucidum* polysaccharides exert a certain promoting effect on the proliferation of mixed lymphocyte cultures induced by mature cells and can induce the rapid initiation of immunity. Fang and Zhong (2002) cultured *G. lucidum* in a triangular container to determine the effect of the initial pH on the biosynthesis of *G. lucidum* polysaccharides and *G. lucidum* acids. Varying the initial pH value between 3.5 and 7.0 has significant effects on cell growth and product biosynthesis. Decreasing the pH from 6.5 to 3.5 gradually increases extracellular and intracellular polysaccharide production. Chen et al. (2004) studied the effect of *G. lucidum* polysaccharides on the expression of cytokines in mouse spleen cells and proposed that macrophages mainly act through the F3 fragment connecting to the TLR4 receptor on the surface of the cell membrane and activating extracellular signal‐regulating kinase function. Related studies have shown the effect of *G. lucidum* polysaccharides on enhancing the activity of immunoreactive cells in immunosuppressed mice and confirmed that a low dose of polysaccharides (2.5 mg/kg) had the most obvious effect, which could accelerate the recovery of immunosuppression in mice treated with cyclophosphamide (an immunosuppressive antitumor drug) without significant side effects (Zhu et al. 2007). This concept provides a basis for research on the immune‐enhancing effects of *G. lucidum* polysaccharides in reducing chemotherapy‐induced immunosuppressive factors in cancer patients. Fu et al. (2019) explored the antitumor effect and mechanism of *G. lucidum* polysaccharides on the tumor cells of mice. The oral administration of *G. lucidum* polysaccharides for 10 consecutive days significantly reduces the mass weight of malignant sarcoma‐180 in a dose‐dependent manner. Moreover, *G. lucidum* polysaccharides also exhibit different cytotoxicity to a variety of cancer cells and inhibit tumor activity. Ex vivo studies conducted in human blood cells (leukocytes, and human peripheral blood lymphocytes) show the radioprotective effect of β‐glucan of aqueous extract of *G. lucidum* against γ‐ray radiation‐induced damage. In plasmids, they can reduce radiation damage as an increment of the open circular form, as well as increase the DNA extension, as shown in vitro studies (González et al. 2020).

### 2.2 Amino Acids and Proteins

*G. lucidum* contains several different amino acids, such as glutamic acid, proline, aspartic acid, etc., which are essential for the human body, accounting for about 2.94% of the total mass (Ahmad 2018). These amino acids provide enough energy to promote the growth of nucleic acids and proteins in the hypha and activate cell regeneration. Different *G. lucidum* have different types of amino acids, like glutamic acid; the content in red *G. lucidum* is almost double that found in common varieties. Girjal et al. (2012) isolated a peptide from the water extract of *G. lucidum*. The structure and molecular weight of the antioxidant properties of the peptide fractions were determined by various methods. The amino acid composition of the peptide was found to be rich in phenylalanine, aspartic acid, proline, histidine, and isoleucine. It was concluded that the beneficial antioxidant properties of polypeptides may be due to their low molecular weight and specific amino acid composition. Zhang et al. (2018) used the Response Surface Methodology with Box–Behnken Design to optimize the amino acid extraction. The amino acid automatic analyzer detected a total of 18 amino acids with a content of 2.94%. In vitro activity tests showed that these amino acids exhibited an obvious glucosidase inhibitory rate, which can arrive at 380.62 μg/mL. These amino acids possessed strong hypoglycemic and antioxidant activities.

Fungal immunomodulatory protein is a new bioactive protein that widely exists in *G. lucidum* and other medicinal fungi. Fungal immunomodulatory protein is like lectin in terms of structure and immune regulation. To date, six such active proteins have been found and identified in *G. lucidum*, and most of these proteins have medicinal functions, such as blood coagulation, anti‐allergy, and antitumor effects (Qu et al. 2018). The oligopeptides in *G. lucidum* also exert obvious anticancer effects, such as inducing a mitochondrial imbalance in cancer cells and thus causing cancer cell apoptosis. In addition, the downregulation of reductases with various properties is also involved in the process of inducing apoptosis in cancer cells (Liu et al. 2020).

### 2.3 Sterols

Sterols have certain medicinal effects, and these metabolites are even considered hidden antitumor promoters and can effectively prevent and cure cancer through inhibition (Akihisa et al. 2007). More than 20 types of sterols are found in *G. lucidum*, and ergosterol accounts for approximately 3‰ of sterols. Mei et al. (2019) isolated three new ergosterols from *G. lucidum*'s fruiting body and studied their structure using nuclear magnetic resonance and mass spectrometry technology. This study showed that these active ingredients have significant inhibitory activity on nitric oxide production. Jeong and Park (2020) studied the effect of ergosterol on obesity and concluded that ergosterol peroxide plays an important role in inhibiting triglyceride synthesis and adipocyte differentiation at the protein and mRNA levels. Hajjaj et al. (2005) found some oxygenated sterols from *G. lucidum* that can inhibit cholesterol biosynthesis via conversion of acetate or mevalonate as a precursor of cholesterol. Xu et al. (2021) isolated the sterols of *G. lucidum*, which were characterized by HPLC‐ELSD to confirm their structure. Surprisingly, they found the sterols are in higher content than the traditional saponification method. Meanwhile, these sterols can inhibit inflammation in macrophages by significantly attenuating LPS‐induced cell polarization and releasing some important cytokines. Ergosterol peroxide could stimulate cell death of a panel of cancer cells, including human hepatocellular carcinoma cells, HepG2. Ergosterol peroxide is a member of a class of fungal secondary metabolites of 5α,8α‐endoperoxide sterol derivatives. The results showed that ergosterol peroxide induced cell death and inhibited cell migration, cell cycle progression, and colony growth of human hepatocellular carcinoma cells. Li et al. (2016) further examined the mechanism associated with this effect and found that treatment with ergosterol peroxide increased the expression of Foxo3 mRNA and protein in HepG2 cells. The upstream signal proteins pAKT and c‐Myc, which can inhibit Foxo3 functions, were clearly decreased in HepG2 cells treated with ergosterol peroxide. The levels of Puma and Bax, pro‐apoptotic proteins, were effectively enhanced. From the aforementioned literature, it was concluded that ergosterol peroxide stimulated Foxo3 activity by inhibiting pAKT and c‐Myc and activating pro‐apoptotic proteins Puma and Bax to induce cancer cell death.

### 2.4 Terpenoids

**FIGURE 1:**
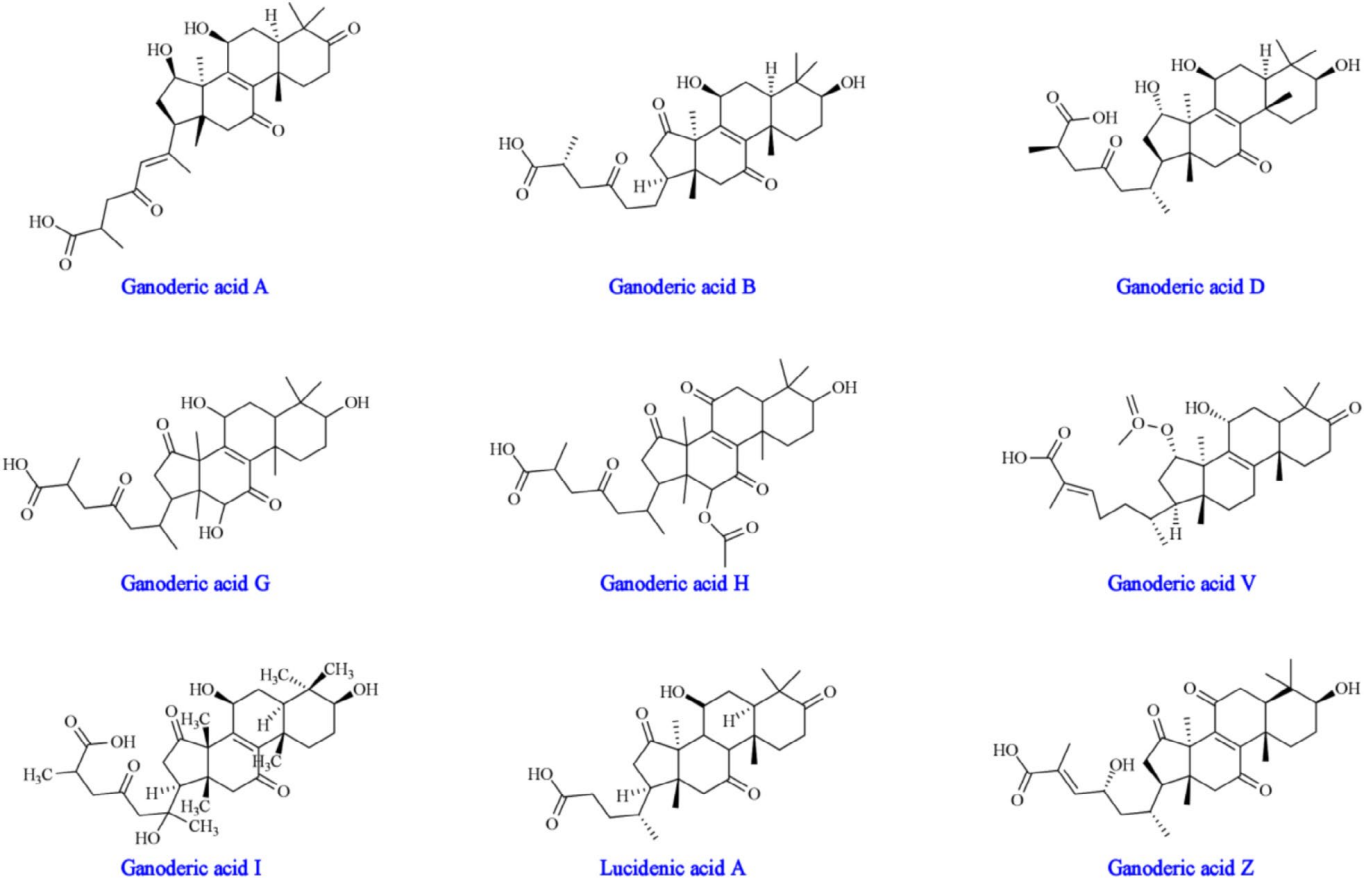
Chemical structures of ganoderic acids. Reproduced from Ref. (He et al. 2023) with permission from Elsevier, Copyright 2023.

Terpenoids are natural products with extremely diversified structures, but only a few of these metabolites have been explored to date (Cao et al. 2022). Till now, more than 300 types of triterpenoids have been isolated from *G. lucidum*. Structurally, triterpenoids constitute a class of highly oxidized lanostane derivatives, predominantly featuring tetracyclic or pentacyclic configurations. These triterpenes exhibit intricate chemical structures adorned with diverse functional groups and distinct side chain variations. Categorized by their carbon atom count, they are delineated into three primary groups: C30, C27, and C24 (Baba et al. 2024). According to differences in functional groups and side chain structures, triterpenoids include ganoderic acid (Figure 1), ganoderic alcohol, ganoderic aldehyde, ganoderic lactones, and others (Shao et al. 2016).

Ganoderic acids, triterpenoids derived from *Ganoderma lucidum*, typically possess a relative molecular mass within the range of 400 to 600 Da (Lian et al. 2024). Characterized by their high liposolubility, they resist dissolution in water (Wu, Zhang, Yin, et al. 2024). With a predominantly bitter flavor, their intensity of bitterness frequently mirrors their concentration.

**FIGURE 2:**
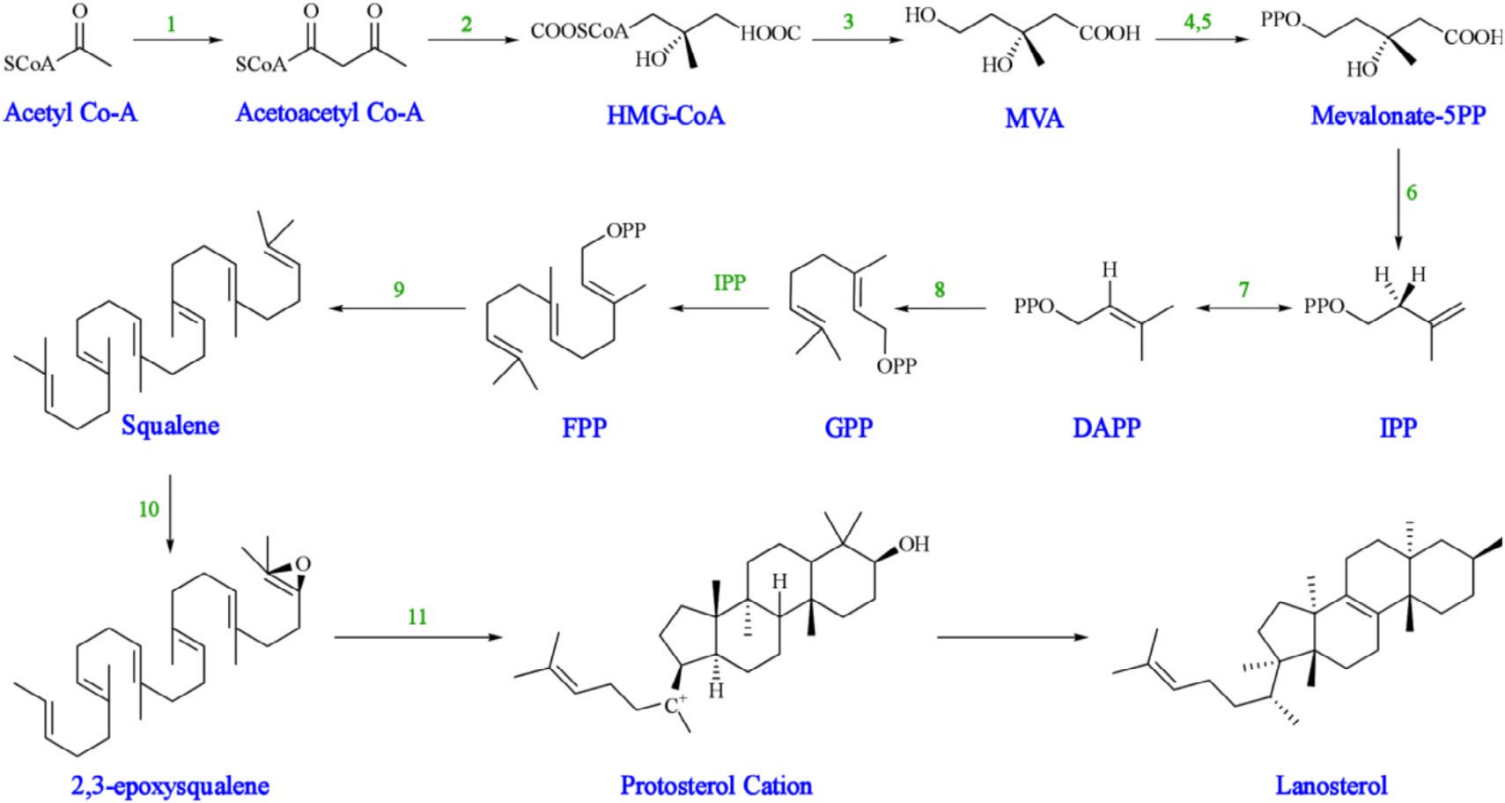
Outline of the MVA and lanostane‐type triterpenoids biosynthesis (Galappaththi et al. 2023). Enzymes involved in the pathway are: (1) Acetyl‐CoA acetyltransferase; (2) 3‐Hydroxy‐3‐methylglutaryl‐CoA synthase; (3) 3‐Hydroxy‐3‐methylglutaryl‐CoA reductase; (4) Mevalonate kinase; (5) Phosphomevalonate kinase; (6) Phosphomevalonate decarboxylase; (7) Isopentenyldiphosphate isomerase; (8) Farnesyl diphosphate synthase; (9) Squalene synthase; (10) Squalene monooxygenase; (11) 2,3‐Oxidosqualene‐lanosterol cyclase.

These triterpenoids can be isolated from Ganoderma's fruiting bodies, cultured mycelia, and basidiospores (De Oliveira Campos et al. 2024; Galappaththi et al. 2023). Ganoderma triterpenoids are biosynthesized via the isoprenoid pathways, specifically the Mevalonate pathway (Figure 2)—a vital metabolic route comprising four main processes: conversion, construction, condensation, and post‐modification. Initially, acetyl‐coenzyme A transforms into isopentenyl pyrophosphate. Subsequently, various prenyltransferases yield farnesyl pyrophosphate, geranyl pyrophosphate, and geranylgeranyl pyrophosphate—advanced terpenoid building blocks derived from isopentenyl pyrophosphate. These mediators can self‐condense and participate in alkylation to produce prenyl side chains (for diverse nonterpenoids) or form rings (constructing triterpenoid skeletons). Ultimately, oxidation, reduction, conjugation, isomerization, and other secondary reactions amplify triterpenoid's unique characteristics (Galappaththi et al. 2023).

Ganoderic triterpenoids exhibit a plethora of biological and pharmacological activities, which exert inhibitory effects on both Gram‐positive and Gram‐negative bacteria, as well as fungi (Raza et al. 2024). Certain components possess the ability to quell the HIV‐1 virus and protease. By influencing the mitochondrial function of cancer cells, they can induce apoptosis (Gill et al. 2018), thereby protecting the liver and ameliorating liver cell degeneration. Ganoderic triterpenoids’ antioxidant prowess surpasses that of ganoderic polysaccharides. Furthermore, they stimulate the immune response by modulating the expression of interleukin‐6 and tumor necrosis factor (Zheng et al. 2022), exhibiting immunomodulatory and apoptotic‐inducing activities against lung cancer. Notably, they also demonstrate substantial efficacy in reducing blood lipids and blood sugar levels, exhibiting anti‐inflammatory properties. Especially, the ganoderic acids showed significant biological activities (Ansari et al. 2022). Among them, Ganoderic acid DM (50) displayed stronger 5 α‐reductase inhibitory activity (IC 50 value of 10.6 μM) than the positive control (α‐linolenic acid, 116 μM). Meanwhile, compared to its methyl derivative, the inhibitory activities of 5 α‐reductase at 20 μM were 55% and 3% for 50 and its derivative, suggesting that the carboxyl group of the side chain of 50 is essential to elicit the inhibitory activity (Galappaththi et al. 2023). Also, a research showed that Ganoderma tropicum can effectively address coronary heart disease and liver protection and promote restful sleep. Chemical analysis of its fruiting bodies unveiled a nortriterpenoid, designated 26‐nor‐11,23‐dioxo‐5α‐lanost‐8‐en‐3β,7β,15α,25‐tetrol. Tests were conducted to assess compounds 1 and 2's inhibitory activity against acetylcholinesterase, and it revealed a minor inhibition rate of less than 10% at a concentration of 100 μM, suggesting an insignificant impact on acetylcholinesterase inhibition (Hu et al. 2014). Viet Hung et al. (2022) conducted an exhaustive study on the fingerprint profiles of five distinct strains of *G. lucidum* , dating from Japan, Korea, China, and Vietnam. Additionally, they examined five samples of *G. lucidum* thriving on *Erythrophloeum fordii* Oliv. in Vietnam, as well as five related Lingshi species, namely, Ganoderma applanatum, Ganoderma australe, Ganoderma clossum, Ganoderma subresinosum, and Ganoderma sp., utilizing high‐pressure thin‐layer chromatography to delve into their triterpene derivatives. Results unveiled marked distinctions between different *G. lucidum*, particularly in the realm of triterpene derivatives. An assessment of the cytotoxicity of these samples against four cancer cell lines—A549, MCF7, PC3, and HepG2—revealed diverse cytotoxic impacts. Specifically, IC 50 values ranged from 15.6 to 46.3 μg/mL for A549, 18.4 to 43.6 μg/mL for MCF7, 10.0 to 32.1 μg/mL for PC3, and 10.6 to 27.6 μg/mL for HepG2. Zhao et al. (2025) isolated bioactive triterpenes from *G. lucidum*, subsequently utilizing experimental mice to assess their anti‐HCC efficacy in vivo. Through lncRNA array analysis and qRT‐PCR validation, six pivotal lncRNAs emerged from the lncRNA‐mRNA co‐expression networks. Bioinformatics insights unveiled FR036820 and FR121302 as crucial en‐lncRNAs in hepatocellular carcinoma, modulating the adjacent genes Ttc22 and Popdc2, respectively. In vitro experiments corroborated that FR121302‐Popdc2 could constitute a core pathway in *G. lucidum* 's antitumor mechanism. Furthermore, a stable FR121302‐shRNA and overexpressing tumor cell line confirmed FR121302's role as an enhancer RNA, regulating Popdc2 expression in hepatocellular carcinoma. Furthermore, Kou et al. (2022) delved into ganoresinoid A, which potently curbs nitric oxide, IL‐1β, IL‐6, and TNF‐α levels in LPS‐activated BV‐2 microglial cells by suppressing the TLR‐4/NF‐κB and MAPK signaling cascades. Concurrently, this compound markedly mitigated LPS‐induced apoptosis through the reduction of mitochondrial membrane potential and reactive oxygen species. Moreover, ganoresinoid A exhibited antioxidant prowess in H_2_O_2_‐induced SH‐SY5Y cells by activating the Akt/GSK‐3β/Nrf2 signaling axis.

Various extraction methods have advantages and disadvantages. Therein the solvent extraction method has a lower yield and longer production cycle. The extraction of terpenoids from *G. lucidum* is of great significance to the exploitation and utilization. When extracting Ganoderic triterpenoids, their liposolubility necessitates the use of solvents like ethanol and methanol for extraction or reflux (Tang et al. 2016). However, this process is frequently impeded by factors such as solvent type, duration, and temperature. Ultrasonic‐assisted extraction for Ganoderma triterpenoids harnesses cavitation and stirring to bolster extraction rates, conserving solvents and slashing time (Shen et al. 2020). Conversely, microwave‐assisted extraction leverages high‐frequency electromagnetic waves to penetrate cell structures, ensuring swift and energy‐efficient extraction (Bhadange et al. 2024). Lastly, supercritical fluid extraction employs supercritical CO_2_ as a benign, non‐toxic solvent, operating at low temperatures to safeguard heat‐sensitive components (Herzyk et al. 2024). Hua et al. (2025) aimed to refine the continuous reflux extraction of triterpenes from *G. lingzhi* via orthogonal testing, and the optimal conditions were achieved and verified. The optimal parameters comprised an ethanol concentration of 80%, an extraction duration of 1.5 h, and a solid–liquid ratio of 1:26. The resultant average extraction yield stood at 2.412%, with a relative standard deviation of 1.079%. Utilizing the inhibition rate of α‐glucosidase activity as a hypoglycemic activity benchmark, *G. lingzhi* triterpenes demonstrated a notable hypoglycemic effect. Presently, advancements have been made in the extraction and analysis of ganoderic acids, yet their full mechanisms of action remain elusive. Large‐scale, efficient extraction technologies await further refinement. Hence, future endeavors must delve deeper into these mechanisms and enhance technologies, fostering their industrial application across diverse fields.

### 2.5 Alkaloid

**FIGURE 3:**
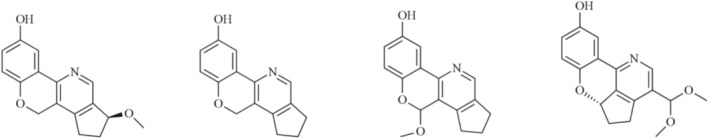
Structures of phenyl‐substituted cyclopenta clpyridine derivatives (Type VI) (Zhang et al. 2022).

Alkaloids, nitrogenous heterocyclic metabolites, distinguish themselves through their structural diversity and robust bioactivity. They occupy a pivotal role in the realm of organic chemistry and are indispensable in the discovery of synthetic drugs (Zhang et al. 2022). *G. lucidum* harbors a modest quantity of these compounds (Table 1), yet they wield remarkable influence in safeguarding the liver, ameliorating cardiovascular conditions, diminishing cholesterol, and reducing blood lipids. Foremost among them are choline, betaine, nicotinic acid, ganoderine A, and ganoderine B. Though their concentration in *G. lucidum* pales in comparison to other active agents, specific alkaloids boast vital biological functions. Figure 3 delineates the structures of select alkaloids. Zhang et al. (2022) summarized seven promising drug candidates, 90 natural products, 37 synthetic compounds, and 26 crucial intermediates. They classified five types of monoterpene pyridine alkaloids and a single type of cyclopenta[c] pyridine alkaloids. Furthermore, they proposed plausible genetic pathways. Subsequently, they delved into the chemical and biotransformations, syntheses, and bioactivities of MTPAs and cyclopenta pyridine derivatives, and cyclopenta pyridine derivatives can be efficiently and chirally synthesized, exhibiting potential in antibacterial, insecticidal, antiviral, anti‐inflammatory, and neuropharmacological applications. Chen and Lan (2018) developed the total syntheses of lucidimines B and C, and these alkaloids were isolated from *G. lucidum* . Notably, lucidimine B shone as a superior antioxidant, outperforming its counterpart, lucidimine C. Similarly, against MCF‐7 cells, lucidimine B exhibited notable antiproliferative prowess, with an EC50 value of 0.27 ± 0.02 μmol/mL, whereas lucidimine C remained inert. By inducing DNA fragmentation, lucidimine B effectively halted the MCF‐7 cell cycle at the S phase, subsequently diminishing the mitochondrial membrane potential. This groundbreaking study underscores, for the first time, the biological potency of *G. lucidum*‐derived alkaloids, hinting at their potential role in the health benefits ascribed to this nutraceutical treasure. Lu et al. (2019) isolated five novel aromatic compounds, named lucidumins A–E, alongside seven known counterparts, from *G. lucidum*. Bioactive assessments revealed their remarkable neuroprotective effects against corticosterone‐induced PC12 cell damage, with cell viability spanning 69.99% to 126.00%. Additionally, select compounds demonstrated significant anti‐inflammatory activities, inhibiting LPS‐induced nitric oxide production in RAW264.7 macrophages, with IC50 values within the range of 4.68 to 15.49 μM. Li, Liu, et al. (2019) discovered two exceptional alkaloid enantiomer pairs, (±)‐1 and (±)‐2, within the rare Ganoderma species, G. luteomarginatum. These alkaloids shared a phenyl‐substituted cyclopenta[c]pyridine scaffold, predominantly occurring in the Ganoderma genus. This unique chemical signature serves as a pivotal taxonomic marker, distinguishing Ganoderma fungi from others. Chen and Lan (2018) have unveiled the total syntheses of lucidimines B and C, pivotal alkaloids of the inaugural family isolated from *G. lucidum* . They further evaluated these synthetically obtained compounds for their antioxidant and antiproliferative efficacies. Notably, lucidimine B emerged as a superior antioxidant compared to its congener C. Analogously, it demonstrated robust antiproliferative activity against MCF‐7 cells, achieving an EC50 value of 0.27 ± 0.02 μmol/mL. Zhao et al. (2015) investigated the bioactive and novel secondary metabolites from higher fungi; four new polycyclic alkaloids lucidimines A–D (1–4) were isolated from the fruiting bodies of *G. lucidum* , and they describe the isolation and structural elucidation of these alkaloids. However, they only studied the relevant extraction methods and structural verification but did not conduct an in‐depth exploration of the related activities and functions. Currently, technologies like supercritical fluid extraction and high‐speed countercurrent chromatography are under relentless exploration in the realm of extraction and separation, aiming to elevate the purity and yield of alkaloids to new heights. Meanwhile, the intricate dance of alkaloid mechanisms of action demands an in‐depth analysis, relying on the precision of molecular biology techniques to unveil their intricate interaction patterns with cellular targets and signaling pathways. Regrettably, further exploration into the compound's medicinal potential remains undertaken.

**TABLE 1:** Some alkaloids in *Ganoderma*.

No.	Alkaloid	Molecular formula	Source	References
1	Australine	C_14_H_13_NO_4_	*Ganoderma australe*	Zhang, Dong, et al. (2021)
2	Lucidimine A	C_16_H_15_NO_3_	*Ganoderma lucidum*	Zhao et al. (2015)
3	Lucidimine B	—		
4	Lucidimine C	C_16_H_15_NO_3_		
5	Lucidimine D	C_17_H_17_NO_4_		
6	Lucidimine E	C_13_H_11_NO_4_	*Ganoderma lucidum*	Lu et al. (2019)
7	Ganocochlearine A	C_14_H_13_NO_2_	*Ganoderma cochlear*	Lie et al. (2015)
8	Ganocochlearine B	C_15_H_15_NO_2_		
9	Ganocochlearine C	C_18_H_17_NO_3_	*Ganoderma cochlear*	Wang, Dou, et al. (2017)
10	Ganocochlearine D	C_17_H_17_NO_4_		
11	Ganocochlearine E	C_17_H_17_NO_3_		
12	Ganocochlearine F	C_15_H_11_NO_4_		
13	Ganocochlearine G	C_15_H_15_NO_3_		
14	Ganocochlearine H	C_15_H_13_NO_2_		
15	Ganocochlearine I	C_15_H_13_NO_3_		
16	(+)‐6S‐hydroxyganocochlearine A	C_14_H_13_NO_3_	*Ganoderma luteomarginatum*	Li, Liu, et al. (2019)
17	(−)‐6R‐hydroxyganocochlearine A			
18	Sinensine	C_15_H_15_NO_3_	*Ganoderma sinense*	Liu et al. (2010)
19	Sinensine B	C_14_H_13_NO_2_		Liu et al. (2011)
20	Sinensine C	C_14_H_13_NO_3_		
21	Sinensine D	—		
22	Sinensine E	—	*Ganoderma luteomarginatum, Ganoderma sinense*	Li, Liu, et al. (2019); Liu et al. (2011)

### 2.6 Others

*G. lucidum* contains a variety of vitamins, including the vitamins B family and C family, which are indispensable water‐soluble nutrients for the human body. Among them, vitamin B can resist pernicious anemia and play a coenzyme role in the biological body; vitamin C can remove free radicals from oxygen in the body and prevent tooth bleeding. Furthermore, *G. lucidum* contains calcium, magnesium, zinc, manganese, iron, potassium, germanium, selenium, vanadium, nickel, chromium, and other nutrient elements. Most of these elements form elemental organic polymer compounds, which are easy to be absorbed by the human body.

For example, *G. lucidum* has a certain organic germanium, which has many medical benefits (Ekiz et al. 2023); its essence is a sesquioxide, which can combine the pollutants in the body, excess metal (positive ions) to become germanium compounds (Reddeman et al. 2020). Germanium is one of the most prevalent elements in *G. lucidum*, which can be discharged from the body through the excretory system and plays the role of purifying body fluids. Especially, its organic status has significant biological activities such as reducing blood pressure, antibacterial, anticancer effects, and high activity (El Sheikha 2022). Selenium is another important rare element related to liver function and muscle metabolism (Huang et al. 2022). Meanwhile, chromium can promote the utilization of glucose, which enhances the insulin‐stimulated glucose uptake (Fan et al. 2024). Each element is adapted to a specific function, so this may be an important reason for *G. lucidum* to prevent a variety of diseases and enhance the body's immune function.

Besides, *G. lucidum* also contains rich adenosine compounds and their derivatives, which are active substances on the basis of nucleoside and purine (Li, Du, et al. 2022). Both of them have strong pharmacological effects and have important effects on inhibiting platelet aggregation in the body, reducing blood viscosity, improving blood oxygen supply capacity, and accelerating blood circulation (Boh et al. 2007; Zhang, Wang, et al. 2021).

## 3 Medicinal Effects

**FIGURE 4:**
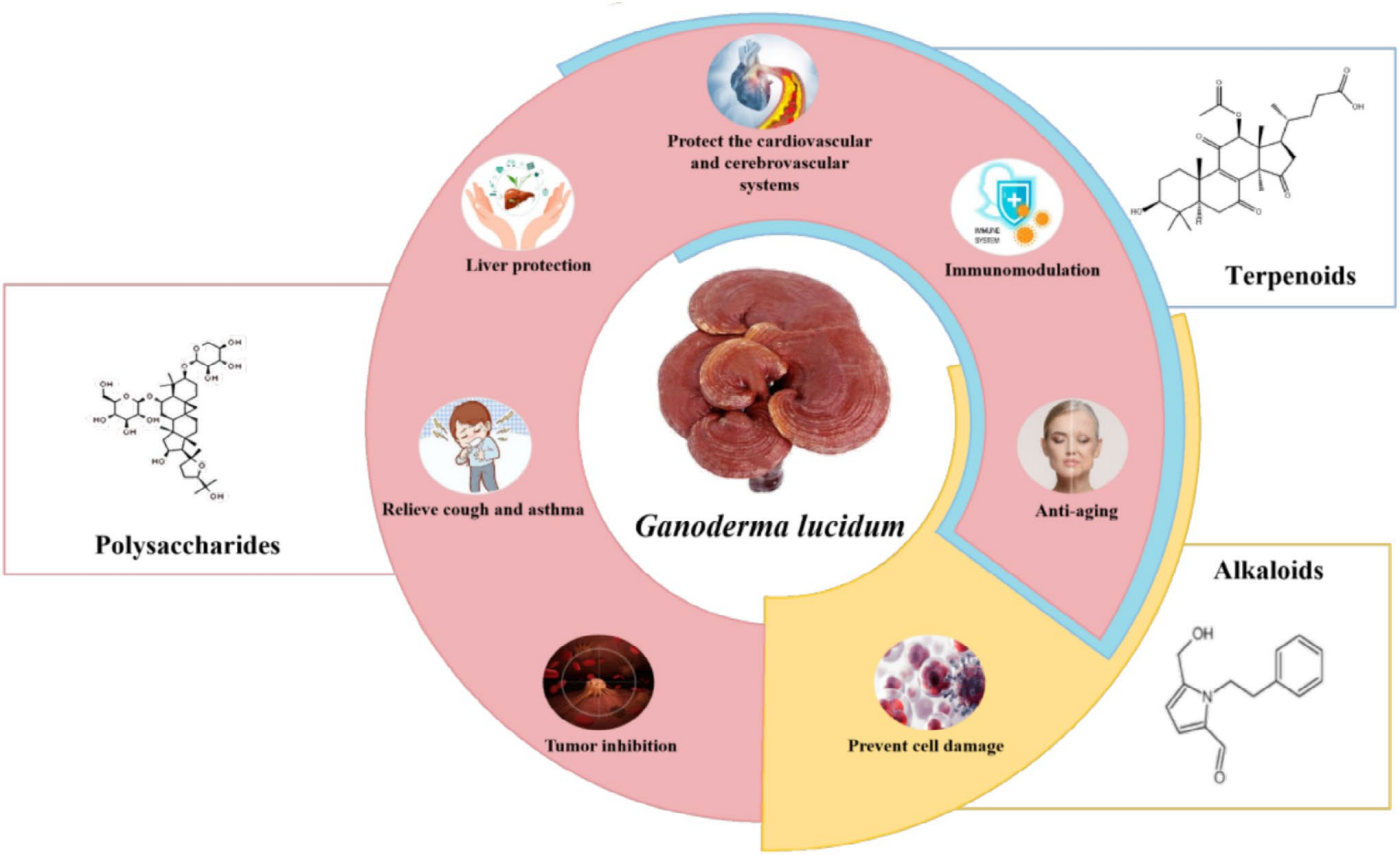
Different pharmacological actions of *G. lucidum*.

The medicinal range of *G. lucidum* is very wide, involving respiratory, circulation, digestion, nervous, endocrine, and movement of various systems, because this fungus can enhance immune function and thus improve body resistance. *G. lucidum* is different from general drugs that only play a certain role in the treatment of a certain disease. It is also different from general nutritional health foods that only supplement the deficiency of certain nutrients but balance the regulation of the body's metabolism. Different pharmacologic actions are presented in Figure 4.

### 3.1 Liver Protection

The incidence of liver injury has been rising in recent years, and its pathogenesis is complex. Oxidative stress and inflammatory stimulation are the main pathological features of liver injury (Ajuwon et al. 2022). Although *G. lucidum* is rich in nutrients and many active pharmaceutical ingredients, which can effectively protect the liver and prevent damage to the liver from external adverse factors (Shieh et al. 2001). The liver is an important metabolic and detoxification organ in the human body. Many factors can induce liver injury, such as abnormal metabolism, drugs, alcohol, viruses, and autoimmune factors. If not treated in time, liver injury can lead to diseases such as fibrosis, cirrhosis, and even liver cancer (Xing et al. 2023). He et al. (2008) investigated the hepatoprotective effects of *G. lucidum*, and they found that the serum aminotransferase levels and activity, as well as the numbers of necrotic and pathological hepatocytes in mice treated with *G. lucidum* extracts were significantly lower than the untreated group. So they obtained the conclusion that CCl4‐induced liver damage can be effectively prevented. However, the most critical hepatoprotective component is *G. lucidum* polysaccharide in its aqueous extracts. *G. lucidum* polysaccharides have a helical three‐order stereoscopic configuration, and their pharmacological activity is closely related to the binding form and stereoscopic configuration of the glycosides between monosaccharides (Liu et al. 2022). *G. lucidum* protects the liver through a broad range of mechanisms that include the modulation of liver Phase I and II enzymes, the suppression of β‐glucuronidase, antifibrotic and antiviral actions, the regulation of the production of nitric oxide (NO), the maintenance of hepatocellular calcium homeostasis, immunomodulatory activity, and scavenging free radicals (Ahmad et al. 2023). In recent years, because of the progress of detection technology, more examples of ganoderan can protect the liver have been obtained. It is mainly because ganoderan can eliminate free radicals in the body and improve the abilities of disease resistance and healing (Muñoz‐Castiblanco et al. 2022). Further study of the signal pathways related to liver disease mediated by oxidative stress can lead to the development of more specific targeted therapies for the treatment of liver disease.

### 3.2 Immunomodulation

*G. lucidum* plays a role in immune regulation through its polysaccharides and terpenoids constituents. Ganoderan not only fights inflammation but also reduces the formation of free radicals and oxidative damage during the occurrence of gastric cancer (Pan et al. 2013). Long‐term administration of *G. lucidum* can improve human immunity (Lin 2005) and resist virus invasion (Zhang et al. 2014). *G. lucidum* polysaccharide can have a certain medicinal effect in vitro and in vivo. Lin and Deng (2019). Meanwhile, its immuno‐modulating effects are extensive, including promoting the function of antigen‐presenting cells, mononuclear phagocyte system, humoral immunity, and cellular immunity (Lin 2005). A Cochrane meta‐analysis showed that patients who had been administered *Ganoderma lucidum* alongside chemo‐/radiotherapy were more likely to respond positively than chemo‐/radiotherapy alone. These trials demonstrated improved immune functions as measured by increased CD3, CD4, and CD8 immune response cells. *G. lucidum* contains beta glucans and other polysaccharides, which stimulate innate immunity and activate host dendritic cells (Xie et al. 2016). However, because the whole process is more complicated, the deeper mechanism of action remains to be studied.

### 3.3 Effect of Relieving Cough and Asthma

*G. lucidum* extract, sourced from the medicinal mushroom *G. lucidum*, demonstrates efficacy in treating chronic bronchitis. Its pharmacological properties include antitussive and bronchodilatory effects, significantly reducing cough and asthma symptoms. By soothing airway irritation and relaxing bronchial smooth muscles, this extract alleviates coughing and facilitates easier breathing. Its use in chronic bronchitis management supports respiratory health, offering a natural and effective therapeutic option. Ruan et al. (2014) studied the effects of *G. lucidum* polysaccharide on the expression levels of glucocorticoid‐induced cytokine receptors and donors in alveolar macrophages of asthmatic rats. They found that ganoderan can down‐regulate the signal expression of alveolar macrophages receptors and donors and alleviate the pathological changes of lung tissue. Through this research, it can be proved that ganoderan can have a better therapeutic effect on asthma. Pi et al. (2014) found allergic asthma is an airway inflammatory disease mediated by the Th2 immune responses. So, they used a polysaccharide purified from a deep culture solution of *G. formosanum*, which can stimulate the activation of dendritic cells and adaptive immune response. In a mouse with allergic asthma, systemic administration significantly inhibited Th2‐mediated bronchial inflammation and the development of airway hyperresponsiveness. Though the animal model used in this study may not fully reproduce the condition in humans, it offers important prospects for the development of this polysaccharide as a preventative agent for allergic asthma.

### 3.4 Tumor Inhibition

Since (宮崎 and 西島 1981) found that branched arabinoxyloglucan isolated in the fruit body of *G. lucidum* possesses the antitumor effect, more and more studies have focused on the structure, function, and activity of related polysaccharides. For example, Wu et al. (2012) found that ergosterol peroxide (5α, 8α‐epidioxiergosta‐6, 22‐dien‐3β‐ol), purified from *Ganoderma lucidum*, can overcome the drug resistance of tumor cells. Xu et al. (2011) isolated and identified *G. lucidum* polysaccharide, which has antitumor activities through immunomodulatory. They found that both in vitro and in vivo studies show the Ganoderma polysaccharides can work in the same way. Furthermore, they also found the polysaccharides can effectively suppress tumorigenesis and inhibit tumor growth by the intervention of cytotoxic effects and anti‐angiogenic actions. However, before it can be widely used, there are still some problems that need to be further explored.

Spores are covered with two hard shells composed of chitin and cellulose, which hinder the absorption of the active ingredients in the Ganoderma spores powder, such as polysaccharides and triterpenes. Therefore, in order to fully release and utilize the effective ingredients in *G. lucidum* spore powder, many effective measures have been taken to remove the cell wall, such as enzymic wall‐breaking, ultrasonic wall‐breaking, temperature, and so forth (Chaiyasut et al. 2010). Ganoderan can inhibit tumor angiogenesis and the growth of tumor cells, and its spore extract exerts a significant antitumor effect in vitro. Shen et al. (2024) emphasized the effectiveness of sporoderm‐broken spores of *G. lucidum* in suppressing the malignant characteristics of hepatoblastoma cells.

Specifically, sporoderm‐broken spores restrain hepatoblastoma tumor growth by inhibiting the O‐GlcNAcylation of RACK1 by multiple signaling pathways, which is a post‐translational protein modification. Furthermore, it also affects processes such as cell growth and migration and is abnormally expressed in a variety of malignant tumors. The Ganoderma spore oil could induce death of cancer stem‐like cells, potentially mediated by the molecule ergosterol peroxide (Xie et al. 2016).

### 3.5 Protection of Cardiovascular and Cerebrovascular Vessels

Cardiovascular disease is a leading cause of death in the world, which seriously threatens human health. The risk of cardiovascular disease is influenced by genetic and environmental factors, and many dietary factors in addition to traditional risk factors are associated with cardiovascular disease (Qi 2012). *G. lucidum* plays a biological role in these diseases through specific signaling molecules and pathways. Additionally, *G. lucidum* polysaccharides can effectively reduce human blood pressure and inhibit renal efferent sympathetic nerve activity. The water‐soluble extract of *G. lucidum* can inhibit platelet aggregation as a mechanism to treat atherosclerosis, exhibits good tolerance, and can improve a variety of cardiovascular risk factors, including blood pressure and blood glucose, triglyceride, and cholesterol levels (Klupp et al. 2016).

*G. lucidum* is rich in fatty acids, which can reduce the viscosity of whole blood or plasma and thereby improve the hemodynamics and local microcirculation. This reduction in viscosity facilitates smoother blood flow, allowing for more efficient nutrient and oxygen delivery to tissues and organs throughout the body. Furthermore, the fatty acids present in *G. lucidum* have been shown to exhibit anti‐inflammatory and antioxidant properties, which can further contribute to maintaining healthy blood vessels and reducing the risk of cardiovascular diseases (Meng and Yang 2019).

The triterpenoids and polysaccharides are the most important medicinal ingredients in *G. lucidum* , which can effectively cure cardiovascular diseases. Li et al. (2021) confirmed this conclusion with a laboratory big‐ear white rabbits as the models. Hsu et al. (2018) did a similar work and revealed the atheroprotective properties and confirmed that the triterpenoids in *G. lucidum* were the most important constituents for curing the disease.

Furthermore, in the pressure‐boosting irradiated cardiomyopathy mice model, one extract of spore oil was confirmed for the modification of cardiac function improvement through the circle RNA‐FOXO3 axis, which is an important pathway associated with heart failure.

### 3.6 Anti‐Aging and Anti‐Fatigue Efficacy

Aging is the result of the accumulation of free radicals produced by tissue and occurs when the degree of damage to the body is higher than the repair ability. *G. lucidum* contains a type of natural antioxidant that can delay aging in a high‐quality manner. Using *Caenorhabditis elegans* as an animal model, studies have found that *G. lucidum* can promote resistance to the oxidative stress of paraquat and the heavy metal Cr^6+^ and significantly extend the lifespan of nematodes. By regulating the signaling pathways of the stress response and lifespan, the anti‐aging mechanism of *G. lucidum* has been further elaborated (Cuong et al. 2019). Other studies have shown that *G. lucidum* spore oil (GLSO) can prolong the average survival time of organisms under oxidative stress damage conditions. GLSO eliminates free radicals and significantly increases the mRNA expression efficacy of copper‐zinc superoxide dismutase (Cu, Zn‐SOD), manganese‐superoxide dismutase (Mn‐SOD), and catalase (CAT). GLSO enhances the activity of SOD and CAT and reduces the level of malondialdehyde (Zhang, Cai, et al. 2021). *G. lucidum* polysaccharides have an important anti‐aging effect. After feeding, the monitoring of several important aging indicators of mice has shown that *G. lucidum* can bring the mouse weight, body temperature, and thymus coefficient close to normal levels. *G. lucidum* polysaccharides can also reduce the content of peroxidized lipids in the mouse liver and improve the vitality of the SOD enzyme and liver peroxidase in red blood cells, indicating that *G. lucidum* has strong antioxidant activity, which can effectively protect the body and prevent aging. Because *G. lucidum* extracts can promote the growth of collagen and accelerate skin metabolism, this fungus is often used in cosmetics. *G. lucidum* extract has antioxidant, antibacterial, and antiviral activities, can maintain the level of skin moisture, restore skin elasticity, inhibit melanin synthesis and precipitation, and remove age spots (Li, Mao, et al. 2019). Cuong et al. (2019) used *G. lucidum* water extract to treat *Caenorhabditis elegans* for detecting anti‐oxidative stress and anti‐aging effects. They found that the expression levels of 2746 genes were significantly changed during the aging process, and about 34 genes were reversed in their expression by the treatment of *G. lucidum* in aged nematodes. Therefore, they concluded that *G. lucidum* could regulate the biological processes in the nematodes through multiple signaling pathways and that the *G. lucidum* water extract can be effective against aging. Lin (2018) reviewed the pharmacological research advances of *G. lucidum* in anti‐aging. It can significantly improve the immunity of elderly mice, and the mechanism of action is most probably because it can facilitate T/B lymphocyte hyperplasia and mixed lymphocyte response. Furthermore, the polysaccharides can delay the aging of cells, organs, and tissues through antioxidant stress, and their mechanism is closely related to the antioxidant and free radical scavenging effects. Wang, Cao, et al. (2017) have done similar research; they reviewed that many extractions in *G. lucidum* have some bioactive components, which have some anti‐aging effects, such as polysaccharides, triterpenes, and peptides, etc. Yang, Zhang, Zuo, et al. (2019) discussed in detail that *G. lucidum* could change pigmentation because of its inhibition of tyrosinase activity and its related protein expression. Besides, it can restrain the ultraviolet B to anti‐aging induced matrix metalloproteinase‐1 expression, which can increase procollagen expression, and it also shows some free radical scavenging ability. In contrast, Luo et al. (2014) focused more on the anti‐fatigue and anti‐hypoxia effects of Ganoderma polysaccharides. Ouyang et al. (2016) focused on the chemotherapy‐related anti‐fatigue that can be alleviated by regulating inflammatory factors and oxidative stress.

### 3.7 Impact on Cellular Metabolism

*G. lucidum*'s active compounds are pivotal in regulating human cellular metabolism. Ganoderan exerts a profound influence on cellular energy metabolism, vital for sustaining cellular life activities (Jung et al. 2018). By enhancing mitochondrial function, ganoderan facilitates efficient adenosine triphosphate (ATP) synthesis, ensuring a steady energy supply to cells (Yang, Zhang, Zhao, et al. 2019). Furthermore, they optimize glucose uptake and utilization by modulating glucose metabolism enzymes, maintaining stable cellular energy levels. For example, *G. lucidum* spores can influence the expression of genes related to glycogen synthesis and lipid metabolism. By increasing the expression levels of genes involved in glycogen synthesis and glucose homeostasis, they reduce blood glucose levels in type 2 diabetic rats. Additionally, they can downregulate plasma triglyceride and total cholesterol levels (Wang et al. 2015). They also regulate lipid metabolism, reducing intracellular triglyceride and cholesterol levels, thereby mitigating fat accumulation and preventing diseases linked to lipid metabolism disorders.

Triterpenoids are indispensable in regulating cellular metabolism, whose potent antioxidant prowess efficiently scavenges intracellular free radicals, safeguards the integrity and fluidity of cell membranes, and ensures the normal physiological metabolism of substances (Li, Liang, et al. 2022). By modulating the activity of pivotal metabolic enzymes, triterpenoids meticulously maintain the balance of material metabolism (Li et al. 2024). Additionally, they interact with cell surface receptors, intricately engaging in cellular signal transduction processes and influencing the complex metabolic webs within cells (Liang et al. 2019). For example, by regulating the nuclear factor‐κB (NF‐κB) signaling pathway, triterpenoids curb the expression of inflammation‐related genes, mitigate the disruptive impact of inflammatory responses on cellular metabolism, and uphold the stability of the cellular metabolic milieu (Hu et al. 2020).

Furthermore, the potent constituents within *G. lucidum* possess the remarkable ability to modulate cellular signaling cascades (Wang et al. 2012). Ganoderan, in particular, can ignite pivotal signaling pathways, including the mitogen‐activated protein kinase (MAPK) pathway (Wang et al. 2019) and the phosphatidylinositol 3‐kinase/protein kinase B (PI3K/Akt) axis (Hsu et al. 2002). These pathways serve as the cornerstone for cellular growth, proliferation, differentiation, and metabolic processes. Through their activation, ganoderan exerts a comprehensive influence on cellular metabolic dynamics, fostering the optimal physiological functioning of cells (Zhong et al. 2022).

### 3.8 Other Curative Effects

*G. lucidum* could effectively alleviate many chronic diseases, such as type 2 diabetes and Alzheimer's disease (Xia et al. 2023). Alzheimer's disease remains an incurable global affliction, which is a serious threat to public safety and a serious problem for patients. Because of the complexity of the disease, there is a lack of effective ways to treat it. Chen et al. (2024) found *G. lucidum* containing various bioactive components, which can postpone the progression of this disease, improve cognitive function, and quality of life. Although this work is only in the early stages of treating the disease, it has fully revealed its application prospects.

Rheumatoid arthritis is a systemic autoimmune inflammatory disease, which is a serious threat to human health. Heo et al. (2023) investigated the effects of *G. lucidum* spores oil in a collagen‐induced rheumatoid arthritis model. GLS oil reduced IL‐6 mRNA expression in response to LPS or TNF‐α treatment in primary cultured chondrocytes, which also reduced LPS‐induced and TNF‐α–induced IL‐6 mRNA expressions, so anti‐rheumatic effects and the pain symptoms can be effectively alleviated.

**FIGURE 5:**
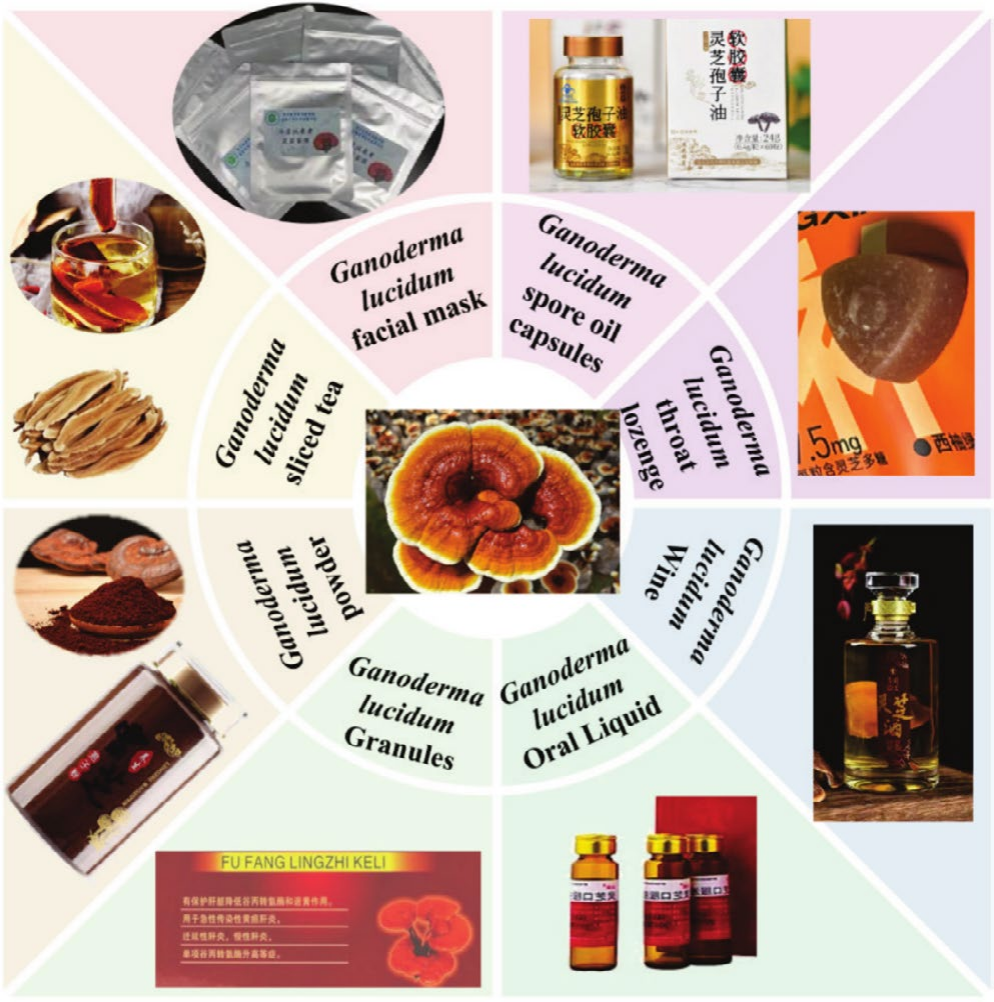
Processing products of *G. lucidum*.

Furthermore, *G. lucidum* has antimicrobial properties. When adding the dried *G. lucidum* powder into smoked fish sausage, the shelf life of which has been extended, it provides an effective way to replace nitrite and other food additives (Wannasupchue et al. 2011). It also has important effects on viruses. Zhang et al. (2014) found *G. lucidum* triterpenoids (lanosta‐7,9(11),24‐trien‐3‐one,15;26‐dihydroxy) and Ganoderic acid Y showed antiviral activities but did not display obvious cytotoxicity in human rhabdomyosarcoma cells. The reason is mostly that the active components can effectively prevent EV71 infection by interacting with the viral capsid proteins to block the adsorption of the virus to the cells. However, the antifungal activity of *G. lucidum* is relatively weak. To delve deeper into its potential antifungal capabilities, the protein ganodermin in the fruiting body of *G. lucidum* was identified as an antifungal protein. Intriguingly, ganodermin inhibits the mycelial growth of various fungi, including *Fusarium oxysporum*, *Botrytis cinerea*, and *Physalospora piricola*, albeit at varying degrees of inhibition concentrations (IC values) (Ahmad et al. 2024). Furthermore, *G. lucidum* has also proven to have a medicinal effect on COVID‐19, which can inhibit the COVID‐19 virus. This antiviral effect has been confirmed by experiments, but because of the pathogenic mechanism of the virus and the target of the medicinal ingredients of *G. lucidum*, there are still many unknowns, so further research is still needed (Wu, Zhang, Peng, et al. 2024). For this reason, *G. lucidum* has been developed into a variety of health products (Figure 5).

## 4 Conclusion

Since 2019, the outbreak of the coronavirus disease (COVID‐19) has sparked a heightened interest in health foods and medications, significantly boosting public health awareness. The effectiveness of *G. lucidum* has garnered widespread recognition. Its active ingredients have been harnessed across various industries, including pharmacology, nutritional health products, and cosmetics. However, despite its widespread applications, there remains a lack of a unified evaluation criterion for its efficacy.

The proven benefits of *G. lucidum* in clinical treatment for chronic diseases necessitate long‐term use, posing a significant challenge in terms of toxicity assessment. As research progresses and detection technologies advance, more *G. lucidum*‐based products are expected to be developed that will further enrich and benefit human life.

The complex chemical composition and medicinal mechanism of *G. lucidum* still remain a mystery. Consequently, its full medicinal value and therapeutic potential await further exploration. Some effective work is actively working to uncover the underlying mechanisms of its action and full potential in treating various diseases. The future holds promise for the continued advancement of *G. lucidum*‐based products.

## Author Contributions

**Sizhu Ren:** writing – original draft. **Wenjie Chen:** writing – review and editing. **Hua Liu, Qing Sang:** data curation. **Mengqi Lu, Qing Gao:** software (equal).

## Consent

The authors have nothing to report.

## Conflicts of Interest

The authors declare no conflicts of interest.

## Data Availability

The authors have nothing to report.
